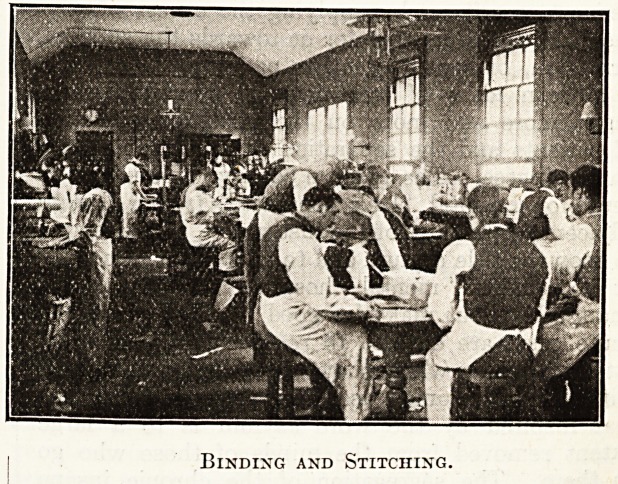# The Work of the Darenth Industiral Colony, Dartford

**Published:** 1913-10-11

**Authors:** 


### THE WORK OF DARENTH INDUSTRIAL COLONY, DARTFORD.

### Dr. A. Rotherham on his Patients' Productivity.

The passing of the Mental Deficiency Act has concentrated attention on the colonies for the feeble-minded and improvable imbeciles which are already in existence. Indeed, as The Hospital recorded a few weeks ago, Boards of Guardians, who have not yet made such provision for this class of patient as is capable of extension and already organised, are paying visits to existing colonies in order to see for themselves on what lines attempts to deal with the feeble-minded have been made hitherto. By the courtesy of Dr. A. Rotherham, the Medical Superintendent, our commissioner was able to pay a special visit of inspection to the Darenth Industrial Colony, and to learn from him how far feeble-minded patients are enabled to contribute to their own maintenance.

### The Economic Factor.

"It is hardly realised," Dr. Rotherham began, "how costly any extended attempt to provide institutional treatment for the feeble-minded and for improvable imbeciles is going to be. The economic factor, however, is of supreme importance owing to the number of persons who may come under these inevitably somewhat loosely defined designations. The inkling of this expenditure ahead of the nation, now that the Mental Deficiency Bill has become an Act, however, may be seen from the number of visits which have been paid by local authorities to Darenth alone, and the object of the visitors has been threefold. In the first place, to learn something of the style of building and planning which experience shows to be desirable in these colonies; secondly, to see the administrative system that is at work here; and thirdly, to learn to what extent the feeble-minded are capable of contributing towards the cost of their support. It will be convenient to walk round the various workshops first; you can then see the buildings and something of the administration; and after the work done has been inspected, I will answer any

Brush and Rug Making.

questions that you may care to put to me concerning the economic value of the work which the patients perform."

As Dr. Rotherham led the way to the workshops, or day wards, as they would be called in an institution where no work was performed, he explained that Darenth, like many other institutions under the control of the Metropolitan Asylums Board, was not originally intended for the special work which it now undertakes. The institution, which consists of a training school, the colony, and pavilions for the feeble-minded, was built about thirty years ago, and intended as a training school for imbecile children. The colony was next erected, in 1880. The present site comprises 164 acres.

"This," said Dr. Rotherham, leading the way into an airy ward much like a school-room in appearance, with its rows of desks and occupants busily at work at needlework and mechanical knitting, "is the needle-room for the adult female patients. Originally intended for rather fewer, now 130 are employed. You will notice the sixty-four Singer's sewing machines, and also the seven Harrison's knitting machines which are in use; and you will perhaps be surprised at the skill which their use

The Needle-room.

implies in the patients, and also at the interest and willingness with which they work."

### Hours of Work and Discipline.

"What are the hours?"

"From 9 to 12 and from 1 to 5. No inducement is required to keep the patients at work, and they require little discipline in the schoolboy sense of the word. The punishment most dreaded by any of the patients is to be removed from the work-room back to the ward. The feeble-minded, moreover, differ markedly from those in full possession of their faculties in two respects which affect administration. They are not prone to play the fool, like schoolboys, or to be careless and to scamp their work, like men who have grown beyond the stage of school larking. Once they show signs of taking an interest in what they do, their patient persistence, industry, and care in the use of mechanical devices, like the knitting machines that you saw just now, are very striking. They like to be at work all day; and though they work more slowly than healthily developed people, they are more steady and sure than most, and it is very rare indeed for us to have an accident with any of the machinery that we use."

"This more resembles a factory than a mental hospital?"

"Exactly. The work is necessarily run on commercial lines, with order-books, elaborate accounts, and so on. The factory inspector pays us periodical visits, and though our products are not intended for the general market, we supply the largest part of what is needed for the forty-seven institutions of the Board. The workshops include those of the tailors, the shoemakers, the upholsterers, the basket-makers, the carpenters, the bookbinders, the printers, the brush-makers, the wood-choppers, the mat-makers, a small tinsmith's, where twenty-one boys are employed, who last year made 3,581 new articles and repaired 345. The workshops for feeble-minded girls include weaving, straw-hat making, and rug making, and in the training school a variety of occupations are taught, like flower work, macramé work (so beloved of English cottagers), Teneriffe and drawn-thread work, and so on. Some of the Teneriffe work is exquisitely done, as also is the sewing, for which certain patients show a wonderful

Printing the Board's Circulars.

aptitude. You will notice that the patients are almost all smiling and cheerful."

The Works Department of the M.A.B."

"How is the work distributed?"

"The Board has a central store in London to which many of the articles are sent, and frequently orders arrive from one or other of the Board's institutions: 'Can you let us have 100 mattresses at once?' and so on. Only a few days ago an order came for 30, and they went off the next morning. Though a certain amount of machinery is employed, for the most part the work is done by hand, and some perhaps would say that there is an irony in the fact that in the twentieth century hand work on a large scale survives mainly among the feeble-minded. How skilled much of their work is can be seen, from another point of view, in the fact that an exhibition of industrial occupations is being held during October in London, for which many articles, furniture among them, are being made here. Indeed, the work of this colony and its function in relation to the other institutions controlled by the same authority may be best described by calling Darenth the Supply Department of the Metropolitan Asylums Board. Innumerable forms, papers, ledgers, bed cards are necessary for its work, and the largest part of the printing of these is done here. We even print our own Prayer-books. The chaplain, Mr. C. M. Jenkins, finding that a shortened form of service was more suitable to the needs of the patients, obtained permission from the Bishop slightly to curtail the established use for morning and evening prayer, and this is now printed and bound here for use in the chapel services."

The Training of Attendants.

"The attendants and nurses have to be specially trained?"

"Yes; in addition to their mental training the nurses are apprenticed for two months to brush-making or whatever may be the occupation in which they intend to become instructors. The industrial attendants, as the men are called, have additional leave, and their hours of work are less than those of the nurses—on the male side from 6 A.M. to 6 P.M. My experience has gone to show that it does not pay to have very highly skilled instructors—men and women, that is, who are experts in the occupation in which they have to instruct the patients here. The reason is that such skill presupposes an interest in the work which militates against the unfailing patience that is required to teach the feeble-minded, and has been acquired without, as a rule, any experience in nursing. It is very important that the instructors should regard their pupils as patients first of all, and the habit of mind which produces this is that which a training in nursing can best provide. The teaching staff in the second building, known as the adult colony, which was converted from an ordinary mental hospital to its present use in 1904, and is capable of housing 1,166 patients, consists, on the male side, of a craftsmaster, Mr. Bickmore, his assistant, and sixteen industrial attendants, together with a master shoemaker and upholsterer. On the female side, under Miss Ferrier, the matron, are a head sempstress, her assistant, and eleven industrial attendants, together with a kitchen and laundry staff. The colony for adults is in part recruited from the training school, and the record which was kept during that preliminary period indicates the sort of work which they are most likely to do well. Sometimes, of course, a patient is moved from shop to shop before a bent is found in him, and I can think now of one of our best carpenters who for six months seemed to make no progress at all in this work, which he now does with great skill. The fact that the patients know that what they make is going to be of practical use, or, in other words, that they are executing orders and not working merely to pass the time, stimulates them enormously, and gives them a degree of self-respect difficult otherwise to find a means of inculcating. Physical drill has also been invaluable in this respect: they perform the exercises extremely well, and not only their physique,

Basket Making.

but their manners and appearance, have improved immensely from it."

How Much the Patients Contribute.

"What is the financial estimate of this improvement as measured by the patients' productivity?"

"Roughly speaking, it may be said that the patients' work pays for the salaries of their attendants, and in addition contributes £2,000 a year towards the expenses of the colony. This figure is, of course, exclusive of the capital expended. In my report for the year ending December 1912 certain facts are recorded which throw a light upon this point. It is a good year to study from the point of view of economy, because in it the scheme for removing all unimprovable patients from Darenth was at last completed. The feeble-minded from the Board's other institutions, moreover, were removed to Darenth. The result was that the feeble-minded and certified imbeciles are under the same administration here, though their quarters are quite separate, and the two classes are not allowed to mix with each other. The removal of the feeble-minded to Darenth does not, therefore, assist in the institutional classification of others who are segregated under the Board.

Envelope Making.

In fact, at the present time, certain patients who have been sent as certified imbeciles, and are therefore housed with them, show greater mental capacity than some of those who are classified as feeble-minded. Different parts of the country, or rather, different medical men, have inevitably different notions of classifying such patients, because feeble-mindedness and imbecility are loose terms for two too loosely defined diatheses. The new Act may or may not lead to definitions of a more generally accepted nature. It is in the light of these facts that the following table, showing the value of the goods made here by the patients and disposed of during the past six years, must be studied :—

*Value of goods made and disposed of, six years ending* 1912.

| | £ | s. | d. |
|---|---|---|---|
| 1907 ... ... ... ... ... | 4,138 | 4 | 7 |
| 1908 ... ... ... ... ... | 8,353 | 5 | 10 |
| 1909 ... ... ... ... ... | 11,032 | 2 | 6 |
| 1910 ... ... ... ... ... | 8,957 | 17 | 8 |
| 1911 ... ... ... ... ... | 11,887 | 1 | 8 |
| 1912 ... ... ... ... ... | 12,366 | 19 | 6 |

And to illustrate the profit and loss account of the various workshops, that of the bookbinders may be added :—

*Bookbinder's Account.*

| Dr. | £ s. d. | Cr. | £ s. d. |
|---|---|---|---|
| Value of stock brought forward ... ... | 186 12 6 | Value of goods disposed of ... | 436 10 5 |
| Value of new stock ... ... | 336 19 1 | Value of stock in hand ... ... | 205 1 9 |
| Wages and rations ... ... | 77 13 11 | | |
| Balance ... | 40 6 8 | | |
| | £641 12 2 | | £641 12 2 |

Patient labour not charged.

These totals do not include the rent or maintenance of the shops. A correct proportion of the craftsmaster's salary is included. The account with the largest figures is that of the needle-room, which you may add for the sake of completeness :—

*Needleroom Account.*

| Dr. | £ s. d. | Cr. | £ s. d. |
|---|---|---|---|
| Value of stock brought forward ... ... | 2,735 1 11 | Value of repairs and goods disposed of ... | 5,180 7 5 |
| Value of new stock ... ... | 4,432 14 2 | Value of stock in hand ... | 2,710 5 6 |
| Wages and rations ... | 562 4 5 | | |
| Balance ... | 160 12 5 | | |
| | £7,890 12 11 | | £7,890 12 11 |

Patient labour not charged.

"What, then, is the average productivity per patient?"

### The Average Productivity Per Patient.

"That is a question which could be answered only by very elaborate calculations, and which, because of the number of factors involved, would be an estimate rather than an answer. As such, it could have little, if any, finality, and I prefer, therefore, to give you the generalisations which have been put forward by various people, and are still current in a general way. But I must premise even generalisations by remarking that different occupations require different amounts of supervision, and that an estimate not unreasonable for certain kinds of work would be quite misleading if applied to other kinds. In laundry work it has been suggested that about twenty healthy, normal women could do the work which forty-five feeble-minded patients could perform, but on the work of the latter cost of superintendence must be charged. Others have supposed that, taking a very rough average for all kinds of occupations, four or five feeble-minded can do the work of one normal man. On this estimate, apart from the fact that were there no supervision no work would be done at all, each feeble-minded person has been adjudged capable of contributing about

Binding and Stitching.

one-fifth towards the cost of his maintenance. But I must warn you that it is far easier to criticise than to justify such figures, and I give them as estimates that have been made, and not the result of my own investigations. The subject is a very interesting and difficult one, and it will be curious to see if light is thrown upon it after the Mental Deficiency Act has been in force for a few years. I may add that the average weekly cost of each patient, excluding rent or loan, and special or central expenses, was last year 9s. 7½d., of which 3s. 4d. represented the cost of maintenance."

## Figures and Tables

**Figure f1:**
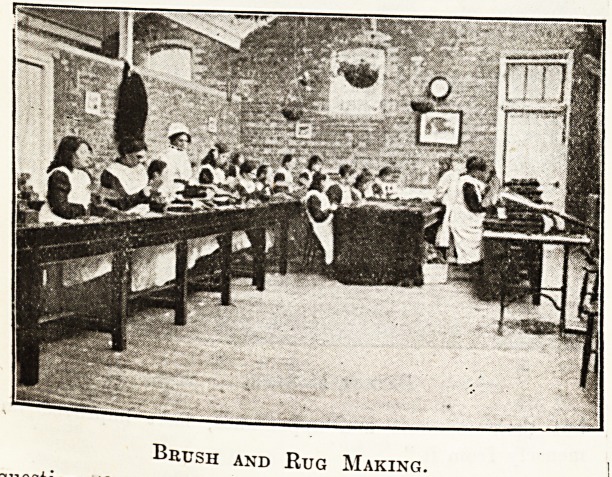


**Figure f2:**
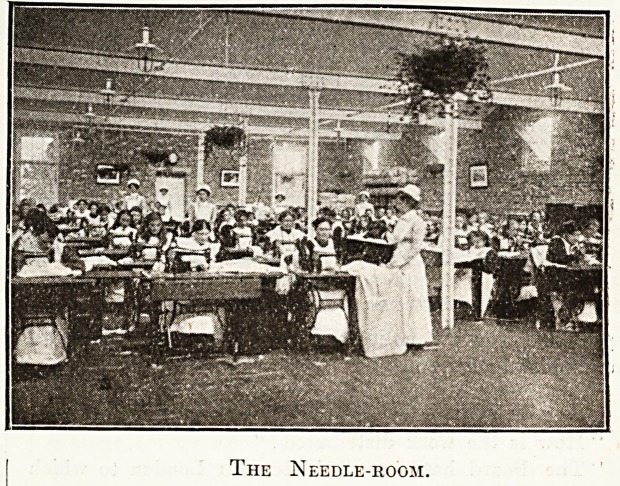


**Figure f3:**
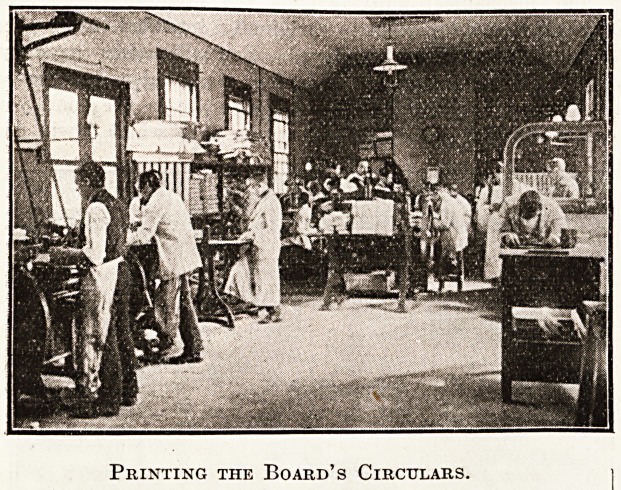


**Figure f4:**
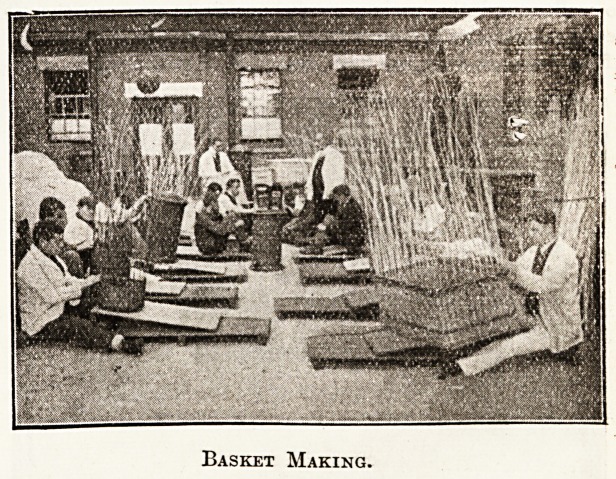


**Figure f5:**
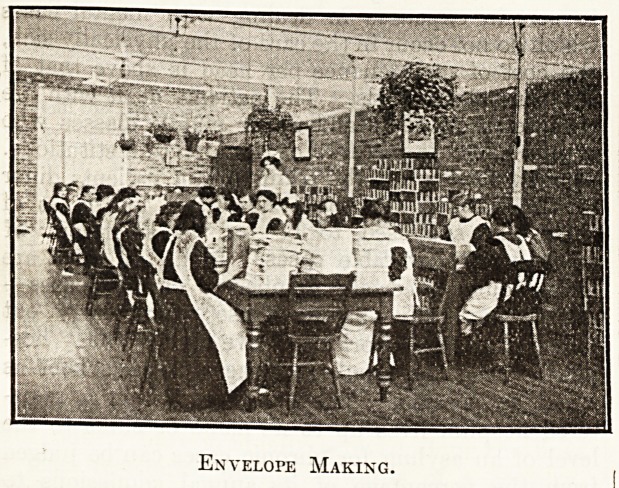


**Figure f6:**